# A Clinical Study on the Efficacy of Acupuncture Treatment in Essential Hypertension: Protocol for Randomized Controlled Trial

**DOI:** 10.2196/71850

**Published:** 2025-05-23

**Authors:** Xiaomin Hao, Lili Zhang, Yu Gong, Jun Wang, Weilan Qin, Xudong Zhang, Qiulei Guo, Meng Xu, Taotao Lv, Yan Guo, Yu Liu, Jipeng Liu, Bingnan Yue, Qingguo Liu

**Affiliations:** 1 Department of Acupuncture and Moxibustion Dongzhimen Hospital Affiliated to Beijing University of Chinese Medicine Beijing China; 2 National Clinical Research Center for Chinese Medicine Acupuncture and Moxibustion First Teaching Hospital of Tianjin University of Traditional Chinese Medicine Tianjin China; 3 School of Acupuncture-Moxibustion and Tuina Beijing University of Chinese Medicine Beijing China; 4 Department of Acupuncture and Moxibustion Dongfang Hospital Beijing University of Chinese Medicine Beijing China; 5 Department of Chinese Medicine Beijing Jishuitan Hospital Beijing China; 6 Department of Rehabilitation Third Affiliated Hospital Beijing University of Chinese Medicine Beijing China; 7 Department of Acupuncture and Moxibustion Beijing Hospital of Traditional Chinese Medicine Capital Medical University Beijing China; 8 School of Special Education Beijing Union University Beijing China

**Keywords:** acupuncture, sham acupuncture, hypertension, randomized controlled trial, study protocol

## Abstract

**Background:**

Primary hypertension represents a significant global public health issue and is a major risk factor for severe cardiovascular disease complications. Previous research has indicated that acupuncture, as a relatively safe therapeutic approach, effectively reduces blood pressure and alleviates clinical symptoms.

**Objective:**

This study aims to assess the clinical efficacy and safety of the “HuoXueSanFeng” acupuncture technique in patients with essential hypertension.

**Methods:**

This investigation is designed as a multicenter, randomized, single-blind, sham-controlled clinical trial. A total of 228 participants with essential hypertension will be recruited from 5 tertiary hospitals in China. Participants will be randomly allocated to either the acupuncture group or the sham acupuncture group in a 1:1 ratio. Each participant will undergo treatment 3 times per week over a 6-week period, amounting to 18 sessions in total. Follow-up assessments will be conducted at 1, 2, and 4 weeks post treatment. The primary outcome measures include 24-hour ambulatory blood pressure and immediate in-office blood pressure readings. Secondary outcome measures included the Dizziness Handicap Inventory, the Headache Impact Test-6, and the Pittsburgh Sleep Quality Index. In addition, any adverse events will be documented throughout the study to assess safety.

**Results:**

This study was registered with the China Clinical Trial Registration Center on May 23, 2024. Data collection commenced in July 2024 and is anticipated to conclude in June 2025. Currently, the study is in the data collection phase, with 27 participants recruited, and data analysis has yet to be conducted. The findings of this study are expected to be submitted for publication in November 2025.

**Conclusions:**

The outcomes of this study are anticipated to further elucidate the benefits of acupuncture in reducing blood pressure and to provide more robust evidence for the treatment of essential hypertension using the “HuoXueSanFeng” acupuncture method.

**Trial Registration:**

Chinese Clinical Trial Registry ChiCTR2400084696; https://www.chictr.org.cn/showprojEN.html?proj=229194

**International Registered Report Identifier (IRRID):**

DERR1-10.2196/71850

## Introduction

Hypertension is one of the major risk factors for fatal complications of cardiovascular disease [1-4] and represents a significant public health concern worldwide. Annually, hypertension is responsible for approximately 10.4 million deaths [5]. Therefore, the primary goals of hypertension management are to regulate blood pressure and prevent damage to target organs [6-8]. In 2015, the global prevalence of adults with elevated blood pressure reached 1.13 billion [9], with projections indicating an increase to 1.56 billion by 2025 [10], thereby posing a considerable global health and socioeconomic challenge [11]. The pathophysiology of essential hypertension is intricate, and its etiology is not yet fully elucidated [12]. Although antihypertensive medications have traditionally been the cornerstone of hypertension management, many patients experience inadequate blood pressure control [13]. Approximately 50% of patients discontinue antihypertensive medications within the first year [14], and adherence rates are generally low [15]. Approximately 9% of patients with hypertension discontinue their medication due to adverse side effects [16]. Furthermore, a significant proportion of patients with hypertension continue to experience severe symptoms associated with hypertension, such as headache, dizziness, and insomnia [17-19]. Consequently, the search for effective and low-harm alternative or complementary therapies has become a critical goal in the management of hypertension [20].

Acupuncture, a traditional Chinese medicine intervention, has been practiced for over 2000 years. Currently, several studies have confirmed that acupuncture is effective in lowering blood pressure and relieving symptoms of hypertension [21-24] with little or only minor side effects [25], making it a relatively safe treatment option [26]. It can serve as an alternative or complementary therapy to lower blood pressure in patients who prefer not to use medication [25], thereby improving adherence to treatment. Acupuncture offers advantages such as multitarget, multipathway, and holistic regulation in the management of hypertension [27]. Nonetheless, the current scientific basis for the modulation of primary hypertension by acupuncture is insufficient [28,29]. There are limited intervention trials comparing acupuncture with sham acupuncture in the regulation of hypertension [24]. Previous clinical investigations have predominantly concentrated on examining circadian rhythm alterations in blood pressure, 24-hour ambulatory blood pressure [25,30-32], as well as the combined use of acupuncture and antihypertensive medications [32-36]. These studies indicate that acupuncture may effectively reduce blood pressure in patients with hypertension. In addition, numerous animal studies have demonstrated that acupuncture significantly reduces blood pressure levels in spontaneously hypertensive rats [37,38]. The underlying mechanisms are complex [27,38,39], and it is noteworthy that studies on the relevant aspects of the onset time, effect duration, and dominant effect of acupuncture in lowering blood pressure remain unclear. Therefore, there is an urgent need for large-scale, well-designed multicenter randomized controlled clinical trials to further investigate the efficacy of acupuncture in treating essential hypertension [36].

Academician Xuemin Shi, a distinguished member of the Chinese Academy of Engineering, is recognized as a pivotal figure in the development of modern acupuncture in China. He pioneered the “HuoXueSanFeng” acupuncture technique, specifically designed for the treatment of hypertension, which is grounded in the “Qihai” theoretical framework. This method involves meticulously defined parameters regarding the direction, intensity, duration, and frequency of the twisting force applied during acupuncture, thereby establishing a standardized and replicable antihypertensive protocol to ensure both the operability and consistency of the treatment.

This study aims to conduct a multicenter randomized controlled trial to evaluate the efficacy and safety of the “HuoXueSanFeng” acupuncture technique in managing essential hypertension. The primary objective is to assess the onset and duration of the blood pressure–lowering effects of acupuncture.

## Methods

### Study Design

This ongoing study is a multicenter, randomized, participant-analyst, single-blind, sham-needle-controlled clinical trial of acupuncture for essential hypertension, conducted in 5 tertiary care hospitals in China. Enrollment will begin in June 2024 and will continue through November 2025. The study will enroll 228 participants with essential hypertension, all of whom will be required to voluntarily sign an informed consent form after rigorous screening. Eligible participants will be randomized in a 1:1 ratio into two groups: the treatment group will be treated with the “HuoXueSanFeng” acupuncture method, and the control group will be treated with sham acupuncture. In this trial, all participants will receive acupuncture 3 times a week for 6 weeks, totaling 18 times. Follow-up visits will be made at weeks 1, 2, and 4 after the treatment.

### Participant Recruitment

This study will be conducted at 5 tertiary hospitals in China: Dongzhimen Hospital of Beijing University of Traditional Chinese Medicine, the First Affiliated Hospital of Tianjin University of Traditional Chinese Medicine, the Beijing Hospital of Traditional Chinese Medicine of Capital Medical University, the Third Affiliated Hospital of Beijing University of Traditional Chinese Medicine, and the Dongfang Hospital of Beijing University of Traditional Chinese Medicine. Participants who meet the inclusion criteria will be enrolled in this study, and we will use 3 ways to recruit participants with essential hypertension. First, we will recruit on site by pasting recruitment posters in the outpatient departments of the hospitals. Second, we will disseminate information about this study via WeChat Moments and the hospital's WeChat Official Account; at the same time, we will distribute posters and hold academic lectures at regular intervals at the 5 hospitals and nearby communities. It is important to note that participants will be excluded from the study design, implementation, and outcome assessment throughout the study. The inclusion and exclusion criteria are detailed in Textbox 1.

**Inclusion and exclusion criteria.**

**Inclusion criteria**

- 18 years old ≤ age ≤75 years old, both male and female individuals.
- Those who meet the diagnostic criteria for primary hypertension in the International Society for Hypertension’s 2020 Practice Guidelines, with a hypertension classification of grade 1 and a risk stratification of low to moderate risk; and those who receive acupuncture treatment while their original treatment is not changed;
- Those who had not taken medication in the past, with an office systolic blood pressure of 140-159 mm Hg and/or a diastolic blood pressure of 90-99 mm Hg; and those who had not changed medication for at least 1 month in the past, with an office systolic blood pressure of 120-159 mm Hg and/or a diastolic blood pressure of 80-99 mm Hg. The types of antihypertensive drugs used are calcium channel blocker+angiotensin converting enzyme inhibitor/angiotensin II receptor antagonist, but no more than two types;
- Correct understanding of the significance of this clinical study, good compliance with the observations and evaluations of the investigators, and voluntary signing of an informed consent form by the participant himself/herself.

**Exclusion criteria**

- Secondary hypertension such as aldosteronism, pheochromocytoma, Cushing’s syndrome, and pregnancy hypertension;
- Patients with other serious cardiovascular, cerebrovascular, renal, retinal, peripheral vascular complications and diabetes mellitus, blood disorders that are not suitable for acupuncture treatment, combined with diagnosed epilepsy, obstructive sleep apnea, hypoventilation syndrome, and so on;
- People with mental disorders such as severe anxiety and depression;
- Pregnant and lactating women;
- Patients who have received acupuncture treatment since the last 1 month or are participating in other clinical trials.

### Randomization and Assignment Hiding

#### Randomization

Randomization will be performed using stratified block randomization. Randomization will be performed by a statistician not involved in trial implementation or statistical analysis, and random sequences will be generated using the Proc plan program in SAS 9.3 software (SAS Institute). Eligible participants will be randomized in a 1:1 ratio into a needling group and a sham needling group. Stratification by center factor will be applied, with block sizes of 4 or 6.

#### Allocation Concealment

The randomization sequence will be kept by a designated person who is not involved in the screening, recruitment, treatment, or evaluation of participants. The randomized grouping scheme is concealed using sequentially numbered, opaque, sealed envelopes, all prepared by staff independent of the study, and after participants sign informed consent and meet inclusion criteria, the envelopes are opened sequentially by the coordinator in the order of enrollment, and the acupuncturist is informed of the grouping in accordance with the allocation scheme inside the envelope.

#### Blinding Setup

The grouping results were kept confidential to the participants, outcome assessors, and data statisticians. Participants received acupuncture treatments in separate compartments of the clinic to avoid interactions and ensure confidentiality. The steps of each acupuncture operation and the method of administration were kept as consistently as possible to minimize bias. Due to the specificity of acupuncture therapy, it was not possible to blind the physician who performed the needling operation. Similarly, outcome assessors and data statisticians were not involved in the experiment throughout, thus controlling for bias that might be introduced to the trial due to knowledge of the allocation scheme. In addition, patients were asked relevant questions at the end of the last acupuncture session to assess the success of the blinding.

#### Acupuncture Group

In the acupuncture group, the original individualized treatment plan will remain unchanged, and the “HuoXueSanFeng” acupuncture method will be used. All therapists have many years of clinical experience in acupuncture.

**Figure 1:**
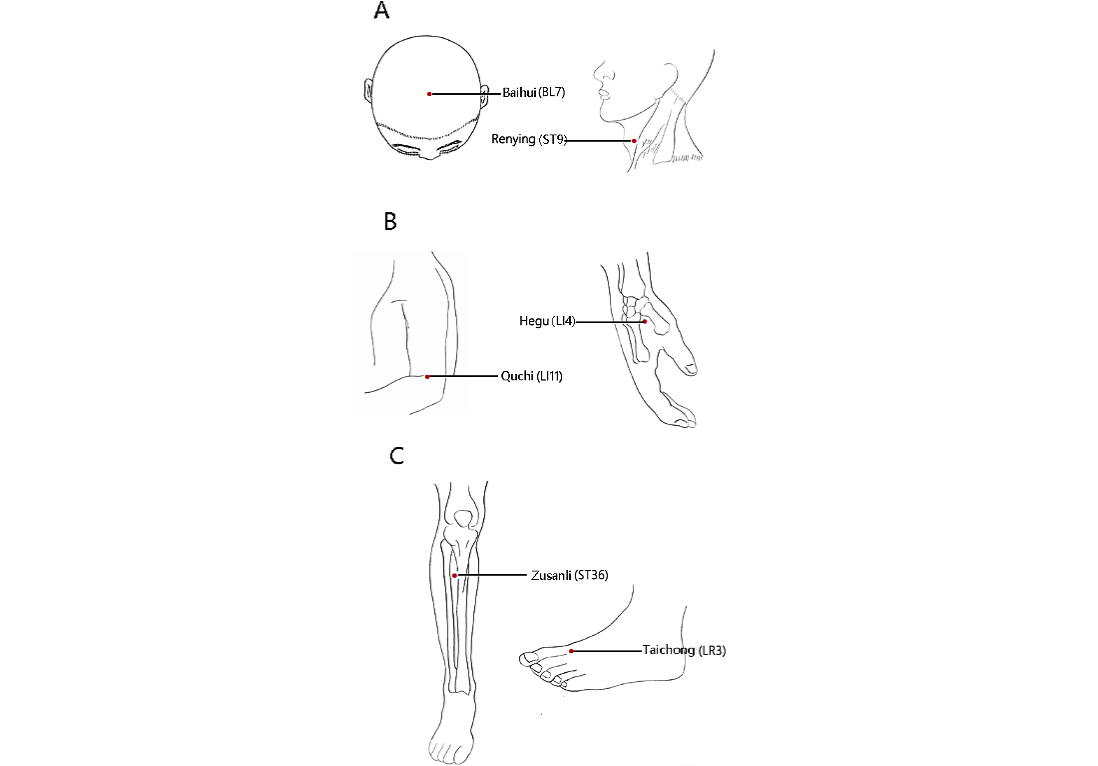
Location of acupuncture points.

Disposable sterile acupuncture needles with a diameter of 0.25 mm and a length of 40 mm (Beijing Zhongyan Taihe Medical Equipment Co, Ltd) will be used. Participants will be placed in a supine position, and the local skin will be routinely sterilized. The acupoints chosen here were bilateral Renying (ST9), Baihui (BL7), Hegu (LI4), ZuSanli (ST36), Taichong (LR3), and Quchi (LI11, right side). The specific localization and operation are shown in Figure 1 and Table 1. The frequency of acupuncture was 120-160 times/minutes, and the twisting amplitude was <90°. Foam pads were fixed on each acupoint, and each acupoint will be administered for 1 minute, after which the needle will be left in place for 30 minutes. The treatment was performed 3 times per week. The measurement time was kept consistent every day, between 9 AM and 11 AM.

**Table 1:** Location of acupoints for the acupuncture group.

Acupoints	Locations	Needling methods
Baihui acupoint (GV20)	On the head, the middle of the front hairline is straight up 5 cun^a^.	Baihui stabbed 10-20 mm along the governor vessel, and then twirled and laxated for 1 min.
Renying acupoint (ST9)	In the anterior part of the neck, the upper edge of the thyroid cartilage (approximately at the level of the laryngeal prominence), the anterior border of the sternocleidomastoid muscle, and the pulsation of the common carotid artery are aligned horizontally.	The needle was inserted vertically for 25~40 mm, and the twirling method was applied for 1 min (both sides of the neck).
Quchi acupoint (LI11)	On the lateral side of the elbow, at the midpoint of the line between the Chize point (LU5) and the lateral epicondyle of the humerus. Quchi point was only punctured on the left side, while the right side was used to measure the immediate blood pressure.	The needle was vertically inserted for 25~40 mm, and then twirled and laxated for 1 min (one side of the left arm).
Hegu acupoint (LI4)	On the back of the hand, between the first and second metacarpals, about the midpoint of the radial side of the second metacarpal.	The needle was vertically inserted for 20 ~25 mm, and the twisting and laxation method was applied for 1 min (both sides of the back of the hand).
Zusanli acupoint (ST36)	On the outside of the lower leg, 3 cun below the Du Bi acupoint (ST35), and on the line between Du Bi acupoint (ST35) and JieXi acupoint (ST41).	The needle was inserted vertically for 25~40 mm, and the twirling method was applied for 1 min (both sides of the lower limbs).
Taichong acupoint (LR3)	In the dorsum of the foot, between the first and second metatarsals, in the depression in front of the metatarsal base junction, or touching the arterial pulse.	The needle was inserted vertically for 20~25 mm, and the twisting and laxation method was applied for 1 min (both sides of lower limbs).

^a^1 cun (≈20 mm) is defined as the width of the interphalangeal joint of the patient’s thumb.

The total observation period was 11 weeks, with a baseline period of 1 week, a clinical treatment period of 6 weeks, and follow-up visits of 15 minutes each at weeks 1, 2, and 4 after the end of treatment.

#### Sham Acupuncture Group

**Figure 2:**
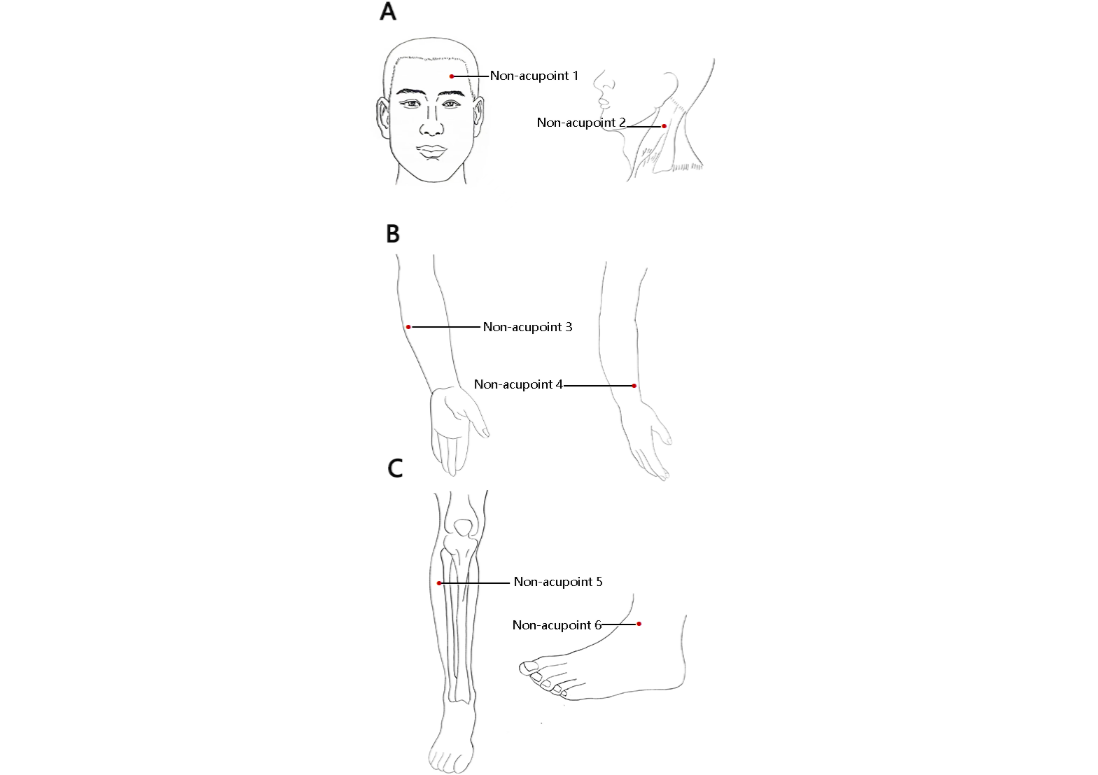
Location of non-acupoints.

Participants in the sham needling group will receive the same duration and frequency of treatment as the needling group, while keeping the original treatment unchanged. Participants will be placed in the supine position and nonmeridian and nonacupuncture points will be selected as the needling location. The specific location and operation method can be found in Figure 2 and Table 2, along with routine sterilization. Foam pads will be fixed on each nonmeridian nonacupuncture point and a disposable sterile comfort needle (customized by Beijing Zhongyan Taihe Medical Instrument Co, Ltd) will be used to pierce through the foam pads to the surface of the skin, simulating needling without actually penetrating the skin, simulating needling without actually penetrating the skin. Participants will be informed that they are receiving a treatment comparing the effects of 2 types of acupuncture to increase their compliance with the treatment while avoiding any deceptive behavior.

**Table 2:** Location of non-acupoints for sham acupuncture group.

Non-acupoints	Locations
Non-acupoint 1	In the middle of Touwei (ST8) and Yuyao (EX-HN4)
Non-acupoint 2	In the neck, between the anterior and posterior edges of the sternocleidomastoid muscle, 1 cun above the Futu point^a^ (bilateral neck)
Non-acupoint 3	On the radial side, the midpoint between the medial epicondyle of the humerus and the first process of the ulna (unilateral on the left arm)
Non-acupoint 4	3 cun above the dorsal stria of the wrist, the midpoint of the line between Pianli (LI6) and Zhigou (SJ6); both sides of the forearm
Non-acupoint 5	Three inches below Yanglingquan (GB34), between the gallbladder and stomach meridians (bilateral lower limbs)
Non-acupoint 6	On the dorsum of the foot, the middle of the connecting line between Qiuxu (GB40) and JieXi (ST41); both sides of the lower limbs

^a^1 cun (≈20 mm) is defined as the width of the interphalangeal joint of the patient's thumb. All non-acupoints were directly punctured with a 1 cun blunt head needle through the fixed pad to reach the skin surface, and evenly lifted, inserted and twisted 3 times each, but the skin was not punctured.

#### Adjustment of Antihypertensive Drug Therapy

In this study, participants are required to maintain their current treatment, including antihypertensive medication, after participating in the trial. This will be documented in detail. Discontinuation of medication, adjustment of medication, and use of over-the-counter or herbal medications will not be permitted during the treatment period.

### Outcome Measurements

#### Primary Outcome Measurement

In this study, the assessor will be blinded to the specific subgroups. The primary outcome will include 24-hour ambulatory blood pressure and in-office immediate blood pressure. We plan to measure 24-hour ambulatory blood pressure once at baseline, once a week 6 of treatment, and once a week 4 after the end of treatment. In-office blood pressure will be measured when the patient first arrives at the office, 5 minutes after lying down, and at 5, 15, and 30 minutes after completing the acupuncture operation and 10 minutes after finishing the needle.

#### Secondary Outcomes

Some of the symptoms associated with essential hypertension, such as vertigo, headache, and insomnia, will be assessed using scales such as the Dizziness Handicap Inventory (DHI), Headache Impact Test-6 (HIT-6), and the Pittsburgh Sleep Quality Index (PSQI). The DHI is a classic tool for assessing vestibular or dizziness-related dysfunction and contains 25 entries covering functional, affective, and somatic dimensions [40]. In hypertensive patients, dizziness is a common symptom that may be associated with blood pressure fluctuations or medication side effects. The HIT-6 is a 6-item questionnaire that assesses the impact of headache on daily functioning in patients with hypertension associated with headache (eg, migraines or medication-induced headache) [41]. The PSQI is a 19-item tool that assesses sleep disorders, which are often comorbid in patients with hypertension (eg, insomnia and sleep apnea) [42,43].

The above assessments will be performed at baseline, at week 6 of starting treatment, and at weeks 1, 2, and 4 after the end of treatment. In addition, other safety indicators will be assessed, including general physical examination, liver and renal function and electrocardiogram at baseline. These tests will be performed at baseline and at week 6 of treatment.

#### Follow-Up

Telephone follow-ups will be conducted at weeks 1, 2, and 4 after the completion of acupuncture treatment. Patients will record their home self-measured blood pressure values, medication intake, and related scale contents accurately and completely in the case report form (CRF). The researcher will provide professional guidance to the patients according to their blood pressure level and related accompanying symptoms.

#### Sample Size and Statistical Analysis

The sample size of this study was estimated using the 2-sample *t* test in PASS (Power Analysis and Sample Size Software; NCSS LLC) 11 efficacy. According to existing literature [44], the mean improvement in systolic blood pressure within 24 hours for patients with essential hypertension treated with acupuncture was 8.9 mm Hg, while the mean improvement for those treated with sham acupuncture was 5.4 mm Hg, resulting in a clinical effect difference of 3.5 mm Hg. The SD for each group was 9.9 mm Hg. The sample size calculation assumed a 2-tailed significance level (α) of 0.05 and a statistical power (1-β) of 80%, the total sample size required for acupuncture treatment of mild-to-moderate essential hypertension was calculated to be 102 cases. Accounting for a 10% dropout rate, a total of 228 hypertensive participants needed to be recruited, with 114 participants in each group.

Statistical analyses we will leave to a statistician who is unaware of the groups. Data will be analyzed using the SAS 9.3 and SPSS 29.0 (IBM Corp) packages, and a 2-sided *P*<.05 will be considered statistically significant. We will use the intention-to-treat analysis set for outcome analysis and also validate it using per-protocol analysis to determine if the two results are consistent and inconsistency will require further analysis and discussion. For patients who withdrew from the study midway through the study, baseline data and follow-up data that had been collected before withdrawal were included in the intention-to-treat analysis. Missing values will be interpolated by the last observation carried forward method.

Baseline data on participants’ demographic information and general state characteristics such as age, gender, and disease duration will be represented by descriptive statistics for both groups. We will measure in-office immediate blood pressure values at different time points of the needle prick and 24-hour ambulatory blood pressure values at baseline, week 6 of treatment and week 4 after the end of treatment, and comparisons between groups will be assessed using repeated measures ANOVA.

For continuous variables, expressed as mean (SD), grouped *t* tests will be used if they conform to normal distribution and vice versa, that is, they will be assessed by Wilcoxon rank-sum test; categorical variables will be compared using chi-square test or Fisher exact test (Fisher), and the results of the data will be documented in the form of percentage.

#### Patient Safety

During acupuncture treatment, participants may experience adverse events such as bleeding, subcutaneous hematomas, persistent feeling of getting gas, increased pain, infection, needle fainting, stress elevation of blood pressure, and allergic reactions to alcohol. As investigators, we will carefully observe changes in the participant’s vital signs and record them in the CRF at all times.

#### Data Management and Quality Control

A thorough assessment will be performed during the screening and preparation phase of the trial and periodically throughout the trial. The screening phase will last for 1 week and will include assessment of basic information such as gender, age, temperature, heart rate, respiration, BMI, etc, as well as detailed information about the participant's chief complaint, current medical history, past history, family history, routine checkups, blood pressure, and medication use. The timing and process of data collection are detailed in Table 3.

Before the formal commencement of the study, all researchers will undergo a uniform and rigorous training to ensure that they are familiar with and equipped with the diagnostic and therapeutic measures and basic assessment skills to improve the consistency of internal observations. According to the study protocol, we will collect complete and authentic raw data from the participants and keep detailed records. Any data that deviate significantly from the clinically acceptable range must be examined and interpreted. The completed and validated CRF will be given to the relevant personnel for data entry, management and statistics, and the data on the CRF will not be modified. Professionals will be assigned to provide regular supervision and quality control throughout the study.

**Table 3:** Clinical research flowchart.

Phase project	Baseline period	On-treatment			Follow-up period		
	Week 0	Week 1	Week 6	Week 7		Week 8	Week 10
Signed informed consent	✓^a^						
Filling in demographic information	✓						
Previous diseases and medication	✓						
Prestudy medication and other treatments	✓						
General physical examination items	✓						
Physical examination	✓						
Acceptance and discharge standard	✓						
24-hour ambulatory blood pressure monitoring	✓		✓				✓
Immediate blood pressure in the consulting room^b^		✓	✓				
Biochemical function test^c^	✓		✓				
Electrocardiogram	✓		✓				
Scale evaluation^d^	✓		✓	✓		✓	✓
Blinded evaluation			✓				
Adverse reactions/adverse events (ADR/AE)	✓^e^	✓^e^	✓^e^	✓^e^		✓^e^	✓^e^
New drug combinations	✓^e^	✓^e^	✓^e^	✓^e^		✓^e^	✓^e^

^a^“✓” in this table represents the items that must be completed; The participants made corresponding records after treatment.

^b^Immediate blood pressure in the consulting room: it needs to be measured in the first to sixth weeks.

^c^Biochemical function test: liver function, kidney function, blood lipid, etc.

^d^Scale evaluation: Traditional Chinese medicine symptom scale, Dizziness Handicap Inventory (DHI), Headache Impact Test-6 (HIT-6), Pittsburgh Sleep Quality Index (PSQI), 36-Item Short Form Health Survey.

^e^Recorded at any time.

#### Dissemination

At the end of the trial, we will endeavor to share the results with other experts from the Chinese medical field, with the aim of benefiting them by exploring the immediate antihypertensive effects of the “HuoXueSanFeng” acupuncture method and its effects on the central nervous system.

#### Biological Specimens

Routine blood tests will be performed and analyzed to collect the necessary data.

### Ethics Approval

This trial was conducted in strict accordance with the Declaration of Helsinki and relevant Chinese clinical trial research norms and regulations. The study protocol has been approved by the Ethics Committee of the Clinical Research Unit (number: 2024DZMEC-153-03) and was successfully registered on May 23, 2024, at ChiCTR2400084696 (Chinese Clinical Trial Registry). Before each participant is enrolled in this study, the investigator will provide a complete and comprehensive written introduction to the purpose, procedures, and potential risks of the study. Participants will be informed of their right to withdraw from the study at any time. If a participant withdraws during the course of the trial, we reserve the right to retain the data obtained for statistical analysis. All data will be anonymized. All participants are entitled to 18 free acupuncture treatments, as this study does not include compensation for transportation and lost wages.

## Results

This study was registered in China Clinical Trial Registration Center on May 23, 2024. Data collection began in July 2024 and is expected to end in June 2025. At present, the data of this experiment is in the collection stage, 27 participants have been recruited, and the data has not been analyzed. The experimental results are expected to be submitted and published in November 2025.

## Discussion

### Anticipated Findings

This study is a multicenter, randomized, single-blind, sham acupuncture-controlled clinical trial designed to assess the efficacy and safety of the “HuoXueSanFeng” acupuncture method in the management of hypertension.

Hypertension is intricately linked with a range of comorbidities, including cardiovascular disease, chronic kidney disease, and diabetes mellitus [45,46]. The primary focus of hypertension management is the regulation of blood pressure and the protection of target organs. Nonetheless, the global burden, health care costs, and societal impact of hypertension are escalating, particularly in low- and middle-income countries [3,47]. Although oral pharmacotherapy remains the cornerstone of hypertension, many patients experience suboptimal blood pressure control, attributable to factors such as the single-target action of medications and their associated adverse effects [48]. In recent years, nonpharmacological therapies have gained recognition for their efficacy in blood pressure reduction, largely due to their minimal or absent side effects, in both developed and developing nations [49]. As a nonpharmacological intervention, acupuncture offers distinct advantages in the treatment of hypertension, owing to its multitarget, multipathway, and holistic regulatory effects [50,51]. Acupuncture can not only regulate hypertensive conditions caused by the imbalance of sympathetic and parasympathetic nervous systems by activating different brain regions, but also regulate neurotransmitters in related brain regions and alleviate autonomic responses, thus exerting antihypertensive effects [27]. However, there are fewer high-quality clinical studies on the treatment of hypertension with acupuncture, and the following three problems exist. First, there are no detailed studies to assess when acupuncture is effective in lowering blood pressure and its duration. Second, there is a lack of effective integration of symptoms accompanying the disease (eg, headache, vertigo, and insomnia). Consequently, the current scientific basis for the regulation of hypertension by acupuncture is insufficient, and the mechanism of antihypertensive by acupuncture needs to be further elucidation.

The “HuoXueSanFeng” acupuncture method, founded by Academician Xuemin Shi based on the “Qihai” theory, is used for the treatment of hypertension. In this method, Renying (ST9) is selected as the main point, supplemented by Baihui (BL7), Quchi (LI11), Hegu (LI4), ZuSanli (ST36) and Taichong (LR3), to treat hypertension. In this study, the Renying (ST9) acupoint is selected as the primary focus, with supplementary points including Baihui (BL7), Quchi (LI11), Hegu (LI4), Zusanli (ST36), and Taichong (LR3), to address hypertension. This methodology integrates the specificity of meridian points with anatomical insights from Western medicine. Furthermore, the approach adheres to academic standards for acupuncture techniques and specifies the precise amount of needling required. This acupuncture therapy is regarded as an environmentally friendly, safe, and effective treatment for hypertension. Consequently, the objective of this research was to thoroughly assess both the immediate and short-term antihypertensive effects of “HuoXueSanFeng” acupuncture, employing sham acupuncture as a control group and using office blood pressure and 24-hour ambulatory blood pressure monitoring. In addition, we evaluated the beneficial effects of the acupuncture method using various assessment tools, such as the DHI, HIT-6, and PSQI, to provide robust, evidence-based support for its application in the treatment of essential hypertension.

It is noteworthy that our trial assessed the immediate effect of acupuncture in lowering blood pressure and the persistence of short-term effects, but the long-term efficacy of acupuncture-such as sustained blood pressure control over months or years remains unproven. Hypertension is a chronic disease that requires long-term management, and the durability of nonpharmacologic interventions such as acupuncture must be rigorously evaluated through long-term follow-up studies. Future studies should prioritize longitudinal designs with 6- or 12-month follow-ups to determine whether repeated acupuncture or intensive treatment is necessary to maintain treatment efficacy.

In addition, there are some noteworthy limitations of our trial. The inclusion of patients with essential hypertension was relatively difficult for us because hypertension, if left untreated, increases the risk of cardiovascular and renal complications [12]. To ensure the safety and reliability of the study, we excluded individuals with severe complications in strict accordance with the inclusion criteria. However, as a double-blind design could not be realized in this experiment, we used sham acupuncture as a control and tried to maintain the consistency of the operation between the 2 groups as much as possible. At the same time, we assessed the effectiveness of blinding in time to achieve a single-blind design. Blinding could not be applied to the acupuncturists because they could perceive the sensation under the needle and visually recognize the needling device during the procedure.

### Conclusions

This study is a multicenter, randomized, single-blind, sham-acupuncture controlled clinical trial. We will apply the “HuoXueSanFeng” acupuncture method proposed by Academician Xuemin Shi, aiming to provide more reliable evidence to support the treatment of essential hypertension. In addition, we seek to explore the advantages of acupuncture in lowering blood pressure and grasp the best opportunity for acupuncture to lower blood pressure.

## Abbreviations

CRF: case report form
DHI: Dizziness Handicap Inventory
HIT-6: Headache Impact Test-6
PASS: Power Analysis and Sample Size Software
PSQI: Pittsburgh Sleep Quality Index
